# When cytology misses and ultrasound warns: the predictive role of BRAFV600E mutation in thyroid nodules

**DOI:** 10.3389/fendo.2026.1803314

**Published:** 2026-03-30

**Authors:** Liu Jie, Cai Wenqing, Zhang Guang, Li Shijie, Fu Qingfeng, Li Kangping, Yu Yuexin, Chen Zhuo, Carla Colombo, Francesco Brucchi, Gianlorenzo Dionigi, Liu Xiaoli

**Affiliations:** 1 Department of Thyroid Surgery, China-Japan Union Hospital of Jilin University, Jilin Provincial Key Laboratory of Thyroid Disease, Jilin Provincial Engineering Laboratory of Thyroid Disease Prevention and Treatment, Changchun, China; 2 Department of Pathophysiology and Transplantation, University of Milan, Milan, Italy; 3 Endocrine Oncology Unit, Department of Endocrine and Metabolic Diseases, Istituto Auxologico Italiano IRCCS, Milan, Italy; 4 Division of General Surgery, Endocrine Surgery Section, Istituto Auxologico Italiano IRCCS (Istituto di Ricovero e Cura a Carattere Scientifico), Milan, Italy

**Keywords:** BRAFV600E mutation, fine-needle aspiration, molecular diagnostics, papillary thyroid carcinoma, thyroid nodules

## Abstract

**Purpose:**

Thyroid nodules with high-suspicion ultrasound features but benign cytologic findings remain a diagnostic challenge due to potential false-negative fine-needle aspiration (FNA) results. The BRAFV600E mutation, a hallmark of papillary thyroid carcinoma (PTC), may improve malignancy risk stratification. This study prospectively evaluated the diagnostic performance of BRAFV600E mutation testing in ultrasound-high-suspicion thyroid nodules with non-malignant cytology.

**Methods:**

A prospective observational study was conducted at the China–Japan Union Hospital of Jilin University between January 2019 and July 2024, enrolling 562 ultrasound-high-suspicion thyroid nodules with non-malignant or indeterminate cytology (Bethesda II–III). Preoperative BRAFV600E analysis was performed using PCR-based methods, and all nodules subsequently underwent surgical excision with definitive histopathology. Associations between mutation status, sonographic patterns, and final diagnoses were assessed through univariate and multivariable logistic regression.

**Results:**

Among 562 nodules, 280 (49.8%) harbored the BRAFV600E mutation. Final pathology confirmed 390 PTCs (69.4%) and 172 benign lesions (30.6%). The BRAFV600E mutation was strongly associated with malignancy (66.2% vs. 12.8%, P < 0.001). On multivariable analysis, BRAFV600E positivity independently predicted PTC (OR = 10.36, 95% CI = 6.18–17.35, P < 0.001). The test showed a sensitivity of 66.2%, specificity of 87.2%, positive predictive value of 92.1%, and overall accuracy of 72.6%.

**Conclusion:**

BRAFV600E mutation testing is a valuable molecular tool for evaluating high-suspicion thyroid nodules with cytologically benign or indeterminate findings. Its strong predictive value enables earlier identification of papillary thyroid carcinoma (PTC), but results should always be interpreted alongside clinicopathologic and imaging assessments to guide optimal diagnostic decisions. In this highly selected cohort, BRAFV600E testing added diagnostic value beyond high-suspicion ultrasound features, improving the distinction between true malignancy and benign mimics in a population with a 69.4% baseline cancer prevalence.

## Introduction

Thyroid nodules are a common clinical finding, and although most are benign, a small subset are malignant. Fine-needle aspiration cytology (FNAC) remains the cornerstone of initial risk stratification and management (1, 2). Ultrasound (US) features such as hypoechogenicity, microcalcifications, irregular margins, and a taller-than-wide shape are well-established indicators of malignancy risk and guide patient selection for FNAC (3).

However, even nodules categorized as benign on cytology (Bethesda II) can occasionally yield false-negative results, particularly when ultrasound or clinical findings are highly suspicious (4, 5). This discordance presents a significant diagnostic challenge, as delayed detection of malignancy may compromise timely surgical management and patient outcomes. In standard protocols, Bethesda II nodules are typically managed conservatively with active surveillance, reserving surgery for malignant, indeterminate, or progressive lesions (6). Yet, certain Bethesda II nodules with worrisome sonographic features – marked hypoechogenicity, solid composition, microcalcifications, irregular borders, or a tall-to-wide shape – or with clinical risk factors such as rapid growth, positive family history, or prior neck irradiation, may still harbor malignancy. In these selected cases, cytology alone may not reliably exclude cancer, prompting interest in adjunctive molecular diagnostic tools (7–9).

The BRAFV600E mutation is the most prevalent genetic alteration in papillary thyroid carcinoma (PTC), particularly in the classic variant. Its presence correlates with more aggressive disease behavior, including extrathyroidal extension, lymph node metastasis, and increased risk of recurrence (5–9). Accordingly, BRAFV600E has emerged as both a diagnostic biomarker and a prognostic indicator in thyroid cancer. Major international guidelines now incorporate molecular testing, including BRAFV600E, as an optional adjunct for cytologically indeterminate nodules (Bethesda III–V) to refine malignancy risk and guide surgical decisions. However, routine testing is not universally mandated and remains variably adopted worldwide due to differences in test availability, cost, and perceived incremental value over high-quality ultrasonography and cytology.

The specific role of BRAFV600E testing in nodules with benign cytology (Bethesda II) but highly suspicious ultrasound features remains uncertain and is not formally defined in current American Thyroid Association (ATA) or European Thyroid Association (ETA) recommendations. Nonetheless, adjunctive molecular testing could enhance diagnostic precision in this gray zone, identifying patients who might benefit from closer monitoring or early surgical intervention (6–9).

At our tertiary referral center, BRAFV600E testing has been progressively integrated into the diagnostic workup of selected high-risk nodules. Under our institutional protocol, FNAC is reserved for nodules with abnormal ultrasound or clinical characteristics. Among these, patients with Bethesda II cytology but high-suspicion C−TIRADS features are routinely offered BRAFV600E testing as an adjunct to ultrasound and cytology, with results discussed in a multidisciplinary conference to individualize management. While some cases proceed to observation, those with suspicious molecular findings are prioritized for surgery. Nodules with indeterminate or malignant cytology (Bethesda III–VI) are managed according to established guidelines.

The aim of this study is to evaluate the clinical utility of BRAFV600E mutation analysis in thyroid nodules with high-suspicion ultrasound features but non-malignant cytological results. Specifically, we sought to determine whether integrating molecular testing improves diagnostic accuracy, facilitates earlier detection of PTC, and enhances risk stratification and management in this diagnostically challenging subset of patients.

## Materials and methods

### Power analysis

Before enrollment, a power analysis was conducted to determine whether the sample size was sufficient to detect statistically significant associations between BRAFV600E mutation status, ultrasound characteristics, and the risk of papillary thyroid carcinoma (PTC). Based on previous literature and pilot data, the estimated prevalence of PTC among cytologically indeterminate (Bethesda II–III) nodules ranged from 60% to 70%, with the BRAFV600E mutation present in approximately 40% to 50% of malignant cases. Assuming a two-sided alpha of 0.05 and a target statistical power (1−β) of 80% to detect a medium effect size (Cohen’s d = 0.4; OR ≈ 2.0 for categorical comparisons), at least 195 subjects per group (mutation-positive vs. mutation-negative) were required.

The final analysis included 562 nodules (280 BRAFV600E-positive and 282 BRAFV600E-negative; 390 malignant and 172 benign by postoperative histopathology), providing sufficient power for binary analysis (malignant vs. benign) and for multivariable logistic regression including multiple covariates such as ultrasound features, cytologic classification, and mutation status. *Post hoc* analysis confirmed that the study had greater than 90% power to detect an odds ratio of 2.0 for associations between BRAFV600E mutation and malignancy, and greater than 80% power to detect significant differences in sonographic characteristics between malignant and benign nodules. The final cohort, derived from an initial pool of over 1,000 surgical patients, was adequately powered for all primary and secondary objectives.

### Patients

This study was approved by the Ethics Committee of the China–Japan Union Hospital of Jilin University (No. 2017022231). In this study, we evaluated BRAFV600E mutation testing in ultrasound−high−suspicion thyroid nodules with cytologically benign or indeterminate results (Bethesda II–III), all of which subsequently underwent surgery. Between January 2019 and July 2024, more than 1,000 patients underwent preoperative fine-needle aspiration (FNA), BRAFV600E mutation testing, and subsequent surgery in the Department of Thyroid Surgery, China–Japan Union Hospital of Jilin University. In this retrospective analysis, only patients who ultimately underwent surgery at our tertiary referral center could be reliably identified and included. Consequently, the study cohort does not represent the full denominator of all Bethesda II–III nodules that underwent BRAFV600E testing during the study period. In routine clinical practice at our institution, many patients with benign cytology and low-risk or negative imaging/molecular findings are managed conservatively and referred back to local hospitals for follow-up. These non-surgical cases were not systematically captured in the present database, which inevitably enriches our cohort for nodules with a higher pretest probability of malignancy. Hospital information systems and archived records from the earlier years of the study did not consistently capture structured data on molecular testing in non-surgical patients, which prevented us from accurately estimating the overall incidence of BRAFV600E positivity and the exact proportion of mutation-positive versus mutation-negative nodules that did not proceed to surgery. All study patients received color Doppler ultrasound examinations, performed either in the thyroid surgery outpatient clinic or the ultrasound department, to identify nodules with imaging characteristics suggestive of malignancy. FNAs were performed after clinical assessment by senior thyroid surgeons, typically together with BRAF mutation testing. In our institutional protocol, BRAFV600E mutation testing is routinely offered for nodules with high-suspicion ultrasound features and Bethesda II–III cytology, where the clinical discrepancy between imaging and non-malignant cytology creates the greatest diagnostic uncertainty.

Surgical decision-making was individualized and based on the integration of ultrasound findings, cytologic category, BRAF mutation status, and patient preference. Written informed consent was obtained from all participants before inclusion. Cytologic diagnoses were classified according to the Bethesda System for Reporting Thyroid Cytopathology (TBSRTC, 2nd edition). For this analysis, only nodules categorized as Bethesda II (benign) or Bethesda III (atypia of undetermined significance/follicular lesion of undetermined significance) were included.

Inclusion criteria were: (1) thyroid nodules classified as Bethesda II or III on cytology, (2) high-suspicion ultrasound features consistent with C−TIRADS criteria, and (3) availability of both BRAFV600E mutation testing and postoperative histopathology. Exclusion criteria included prior thyroid surgery, incomplete cytologic or histologic data, or missing BRAF mutation results. Bethesda IV nodules were not included in this study because, in our practice, these lesions are generally managed either with upfront surgery or, when molecular testing is used, with comprehensive multi-gene panels rather than single-gene BRAFV600E analysis.

A total of 562 nodules meeting inclusion criteria were analyzed: 280 BRAFV600E-positive and 282 mutation-negative. According to final pathology, 390 nodules were confirmed as PTCs and 172 as benign lesions.

### Ultrasound and FNA procedure

Ultrasound imaging was performed using a color Doppler ultrasound system equipped with an L15 linear probe (6–15 MHz). Examinations were independently assessed by two experienced thyroid ultrasonographers (each with more than 10 years of experience). In cases of disagreement, classification was reviewed by a chief or deputy chief physician. At our institution, fine-needle aspiration (FNA) of thyroid nodules smaller than 1 cm was selectively performed in patients with high-suspicion ultrasound features (C-TIRADS 4C–5) in the presence of additional clinical risk factors. These included suspicious cervical lymph nodes, prior neck irradiation, a strong family history of thyroid carcinoma, or imaging findings suggestive of extrathyroidal extension. Routine FNA was not performed for sub-centimeter nodules lacking these high-risk features.

According to C−TIRADS guidelines, features indicative of malignancy included marked hypoechogenicity, solid composition, irregular margins, “taller-than-wide” shape, microcalcifications (<1 mm), and extracapsular extension.

For FNA, patients were positioned supine with shoulder elevation to fully expose the neck. After antisepsis and local infiltration anesthesia with 0.5% lidocaine, 22G needles were used to obtain 3to 4 passes under ultrasound guidance. Residual material from the final pass was collected into EP tubes containing 180 μL cytolytic solution, stored at −20 °C, and later processed for BRAFV600E mutation testing. Thyroid FNA smears were initially interpreted by the on-duty cytopathologist and subsequently reviewed by a senior attending cytopathologist with more than 10 years of experience in thyroid cytopathology. Any discrepancies between the initial interpretation and the senior review were resolved by consensus, and the final agreed-upon Bethesda category was used for analysis. Because this two-tiered review is part of routine clinical workflow, interobserver discordance rates were not prospectively recorded and could not be quantified retrospectively.

### Chinese thyroid imaging reporting and data system

C-TIRADS (Chinese Thyroid Imaging Reporting and Data System) is a standardized ultrasound-based malignancy risk stratification tool specifically developed and validated for the Chinese population. Modeled after the ACR-TIRADS and K-TIRADS systems, C-TIRADS integrates multiple sonographic features– such as solid composition, hypoechogenicity, irregular margins, microcalcifications (<1 mm), and a taller-than-wide shape– each weighted to generate a malignancy score. Scores are assigned to categories (C-TIRADS 1–5), which correspond to increasing malignancy risk and guide recommendations for FNA or follow-up. The system aims to improve diagnostic consistency, reduce interobserver variability, and optimize nodule management in Chinese clinical practice. Ultrasound examinations were independently assessed by two experienced thyroid ultrasonographers, and C-TIRADS categories were assigned according to established criteria. In cases of disagreement, a third senior ultrasonographer adjudicated the final risk category. As this adjudication process is part of routine clinical care, formal interobserver discordance rates were not systematically documented.

### DNA isolation and BRAFV600E mutation detection

Mutation analysis was performed using the ADx-ARMS kit (AmoyDX, Xiamen, China). DNA was extracted from cytologic samples using the ADx Amplification System for Mutation Specificity, following the manufacturer’s instructions. DNA concentration was quantified with a NanoDrop 2000 spectrophotometer (Thermo Fisher Scientific, USA).

Amplification and detection of the BRAFV600E mutation were conducted using a real-time PCR system (SLAN-96, Shanghai Hongshi Medical Technology Co., Ltd.) and an Applied Biosystems PCR platform. The reaction contained 5 μL of DNA template with primers, Taq polymerase, fluorescent probes, nucleotides, and buffers. The thermal cycling protocol was as follows: 94 °C for 5 min (initial denaturation); 15 cycles of 95 °C for 25 s, 64 °C for 20 s, and 72 °C for 20 s (initial annealing); followed by 31 cycles of 93 °C for 25 s, 60 °C for 35 s, and 72 °C for 20 s (extension). All analyses were conducted in triplicate to ensure assay validity.

### Cost coverage for BRAFV600E testing

In this study, patients paid directly for BRAFV600E testing of Bethesda II (benign) nodules. For Bethesda III nodules, testing was also generally self-funded, as molecular profiling for thyroid cytology is not routinely reimbursed by national basic medical insurance. However, in a minority of cases, partial coverage was obtained through supplementary commercial insurance or institutional research funds. Thus, cost considerations were similar for both Bethesda II and III categories, with no systematic difference in test availability or coverage between these groups.

### Final histopathology and blinding

Surgical specimens were first evaluated by the staff pathologist and then reviewed and confirmed by a senior pathologist specializing in thyroid pathology. In diagnostically challenging cases, slides were discussed at a multidisciplinary thyroid conference to reach a consensus diagnosis. The senior pathologist was blinded to BRAFV600E mutation status during histological assessment, and molecular results were correlated only after the final pathology report had been issued.

### Statistical analysis

All statistical analyses were performed using SPSS version 27.0 (IBM Corp., USA). Quantitative variables were compared using the Mann–Whitney U test, while categorical variables were analyzed with the χ² test. Multivariable associations were examined using a logistic regression model. A two-tailed p < 0.05 was considered statistically significant.

## Results

### Study population

Of more than 1,000 patients evaluated during the study period, 637 met the initial eligibility criteria. After excluding 74 cases due to incomplete molecular testing or missing follow-up data, 562 patients (562 nodules) were included in the final analysis. Because detailed data on non-surgical patients were incompletely recorded, we were unable to reconstruct the true incidence of BRAFV600E positivity among all Bethesda II and III nodules evaluated at our institution, or to calculate precise surgery-conversion rates stratified by mutation status comparable to those reported in other series.

Among the included nodules, 280 (49.8%) were BRAFV600E mutation positive and 282 (50.2%) were mutation negative. The median age was similar between groups: 46.5 years (IQR 39–52.7) in the mutation-positive group and 47 years (IQR 39.75–55.25) in the mutation-negative group (Z = −1.298, p = 0.194). Postoperative histopathological examination identified 390 papillary thyroid carcinomas (PTC; 69.4%) and 172 benign nodules (30.6%). There was no significant age difference between the benign and PTC groups (Z = −0.851, p = 0.395). No data were missing for cytology, mutation testing, or surgical pathology.

Of the 562 nodules, 351 (62.5%) were Bethesda II and 211 (37.5%) were Bethesda III on cytology. The malignancy rate was 69.5% (244/351) in Bethesda II nodules and 91.5% (193/211) in Bethesda III nodules (p < 0.001), resulting in an overall malignancy rate of 69.4% (390/562) in this surgically treated cohort.

Within the Bethesda II subgroup, BRAFV600E testing retained a “rule-in” profile, with a sensitivity of 43.84%, specificity of 84.11%, PPV of 86.29%, and NPV of 39.65% for PTC (**Supplementary Table 4**). Although sensitivity was lower than in the combined cohort, PPV remained high, underscoring that a positive BRAFV600E result in an ostensibly benign nodule is strongly suggestive of underlying malignancy.

In the Bethesda II group, 244 nodules were malignant, with 107 (43.9%) BRAFV600E−positive and 137 (56.1%) mutation−negative; among the 107 benign Bethesda II nodules, 17 (15.9%) were BRAFV600E−positive and 90 (84.1%) were negative. In the Bethesda III group, 193 nodules were malignant, including 151 (78.2%) BRAFV600E−positive and 42 (21.8%) mutation−negative lesions, while among the 18 benign Bethesda III nodules, 5 (27.8%) were BRAFV600E−positive and 13 (72.2%) were negative.

### Histologic subtypes of PTC

Among the 390 PTCs, most were papillary thyroid microcarcinomas (≤1 cm; 93%), while non-microcarcinomas accounted for only 7%. Classical PTC was the predominant subtype; follicular-patterned variants (including follicular variant PTC and encapsulated tumors) were less common but were overrepresented among BRAFV600E-negative malignancies on descriptive review. PTC was the only malignant histology identified in this cohort; no cases of follicular thyroid carcinoma, medullary thyroid carcinoma, poorly differentiated carcinoma, or anaplastic carcinoma were observed. Consequently, our data do not allow conclusions about the performance of BRAFV600E testing for non-PTC malignancies, and the assay should not be expected to rule out these entities.

### Comparison by BRAFV600E mutation status

Clinicopathologic and ultrasonographic characteristics by BRAFV600E mutation status are presented in **Table 1**. Sex distribution did not differ significantly between mutation-positive and mutation-negative groups.

**Table 1:** Comparison of clinical features between mutation-positive and mutation-negative patients with benign FNA nodules.

Features	BRAF (+) (n=280)	BRAF (-) (n=282)	Statistic	P
Gender				
Male	38 (13.6)	52 (18.4)	2.476	0.116
Female	242 (86.4)	230 (81.6)		
Age				
<45	133 (47.5)	125 (44.3)	0.570	0.450
≥45	147 (52.5)	157 (55.7)		
<55	227 (81.1)	206 (73.0)	5.112	**0.024**
≥55	53 (18.9)	76 (27.0)		
Size (cm)	0.49 (0.38-0.65)	0.64 (0.45-0.96)	-5.660	**<0.001**
<1	260 (92.9)	220 (78)	24.839	**<0.001**
≥1	20 (7.1)	62 (22)		
US hypoechoic				
(+)	267 (95.4)	245 (86.9)	12.458	**<0.001**
(-)	13 (4.6)	37 (13.1)		
US solid				
(+)	187 (66.8)	163 (57.8)	4.827	**0.028**
(-)	93 (33.2)	119 (42.2)		
US Tall/Wide > 1				
(+)	173 (61.8)	102 (36.2)	36.892	**<0.001**
(-)	107 (38.2)	180 (63.8)		
US <1mm calcification				
(+)	62 (22.1)	64 (22.7)	0.025	0.875
(-)	218 (77.9)	218 (77.3)		
US irregular borders				
(+)	251 (89.6)	197 (69.9)	34.011	**<0.001**
(-)	29 (10.4)	85 (30.1)		
No. atypical US findings				
0	7 (2.5)	25 (8.9)	36.084	**<0.001**
1	8 (2.9)	30 (10.6)		
2	37 (13.2)	51 (18.1)		
3	81 (28.9)	80 (28.4)		
4	120 (42.9)	81 (28.7)		
5	27 (9.6)	15 (5.3)		

Bold values in the table indicate statistically significant P-values.

Ultrasound analysis showed that mutation-positive nodules were significantly smaller than mutation-negative nodules, with median sizes of 0.49 cm (0.38–0.65) versus 0.64 cm (0.45–0.96), respectively (Z = −5.660, p < 0.001). The prevalence of nodules smaller than 1 cm was higher in the mutation-positive group (92.9% vs 78.0%, p < 0.001). Mutation-positive nodules more frequently exhibited hypoechogenicity, solid composition, taller-than-wide shape, irregular margins, and a greater number of suspicious ultrasound features (all p < 0.05). There was no significant difference in the frequency of microcalcifications (<1 mm) between the two groups (p = 0.875).

### Comparison by final histopathology

All 562 nodules underwent surgical resection and were classified as benign or malignant according to postoperative histology (**Table 2**). Univariate analysis showed no significant difference in sex distribution (p = 0.266). PTC nodules were significantly smaller than benign nodules (median 0.50 cm [0.38–0.67] vs 0.71 cm [0.47–1.09]; Z = −6.723, p < 0.001), and the proportion of nodules smaller than 1 cm was higher in the PTC group (92.3% vs 69.8%, p < 0.001).

**Table 2:** Comparison of cytologically benign or indeterminate nodules (Bethesda II–III) clinical characteristics between PTC group and benign group.

Features	Benign nodule (n=172)	PTC (n=390)	Statistic	P
Gender				
Male	32 (18.6)	58 (14.9)	1.237	0.266
Female	140 (81.4)	322 (85.1)		
Age	48 (39-56)	46 (39-53)	-0.851	0.395
<45	77 (44.8)	181 (46.4)	0.130	0.719
≥45	95 (55.2)	209 (53.6)		
<55	123 (71.5)	310 (79.5)	4.293	**0.038**
≥55	49 (28.5)	80 (20.5)		
Size (cm)	0.705 (0.47-1.09)	0.500 (0.38-0.67)	-6.723	**<0.001**
<1	120 (69.8)	360 (92.3)	48.662	**<0.001**
≥1	52 (30.2)	30 (7.7)		
US hypoechoic				
(+)	142 (82.6)	370 (94.9)	22.329	**<0.001**
(-)	30 (17.4)	20 (5.1)		
US solid				
(+)	86 (50)	264 (67.7)	15.904	**<0.001**
(-)	86 (50)	126 (32.3)		
US Tall/Wide > 1				
(+)	52 (30.2)	223 (57.2)	34.684	**<0.001**
(-)	120 (69.8)	167 (42.8)		
US <1mm calcification				
(+)	35 (20.3)	91 (23.3)	0.611	0.434
(-)	137 (79.7)	299 (76.7)		
US irregular borders				
(+)	110 (64)	338 (86.7)	38.080	**<0.001**
(-)	62 (36)	52 (13.3)		
BRAFV600E				
(+)	22 (12.8)	258 (66.2)	135.958	**<0.001**
(-)	150 (87.2)	132 (33.8)		

Bold values in the table indicate statistically significant P-values.

Significant differences were also observed in ultrasonographic features: PTC nodules more often showed hypoechogenicity, solid composition, and irregular margins (all p < 0.05). The rate of BRAFV600E mutation was much higher in the PTC group than in the benign group (66.2% vs 12.8%, p < 0.001).

### Multivariable logistic regression analysis

Variables significant in univariate analysis (nodule size, hypoechogenicity, solid composition, irregular margins, and BRAFV600E mutation status) were included in a multivariable logistic regression model (**Table 3**). BRAFV600E mutation was identified as an independent predictor of PTC, conferring a 10.356-fold higher risk compared with mutation-negative nodules (p < 0.001).

**Table 3:** PTC risk in cytologically benign or indeterminate nodules (Bethesda II–III) by multivariable logistic regression analysis.

Features	B	OR (95%CI)	P
Age≥55	-0.202	0.817 (0.492-1.359)	0.437
Size	-0.545	0.580 (0.235-1.433)	0.238
Size≥1cm	-0.614	0.541 (0.201-1.458)	0.224
US hypoechoic	0.169	1.184 (0.498-2.820)	0.702
US solid	0.423	1.527 (0.954-2.445)	0.078
Tall/Wide > 1	0.329	1.390 (0.856-2.255)	0.183
US irregular borders	0.333	1.395 (0.749-2.597)	0.294
BRAF mutation	2.338	10.356 (6.181-17.349)	**<0.001**

Bold values in the table indicate statistically significant P-values.

### Diagnostic performance of BRAFV600E testing

The diagnostic performance of the BRAFV600E mutation assay was as follows: sensitivity, 66.15%; specificity, 87.21%; positive predictive value, 92.14%; and negative predictive value, 53.19%, yielding an overall diagnostic accuracy of 72.60%. Incorporating BRAFV600E mutation analysis into the evaluation of cytologically benign and indeterminate nodules (Bethesda II–III) increased the detection rate of PTC by 41.99% compared with cytology alone (**Tables 4**, **5**).

**Table 4:** Comparing BRAFV600E mutation testing results with postoperative pathologic findings in Bethesda class II subgroups.

Variable	PTC	benign nodule	Sensitivity	Specificity	PPV	NPV	Accuracy
BRAF^V600E^ (+)	107 (43.9)	17 (15.9)	43.85%	84.11%	86.29%	39.65%	56.13%
BRAF^V600E^ (-)	137 (56.1)	90 (84.1)					

**Table 5:** Comparing BRAFV600E mutation testing results with postoperative pathologic findings.

Variable	PTC	benign nodule	Sensitivity	Specificity	PPV	NPV	Accuracy
BRAF^V600E^ (+)	258 (66.2)	22 (12.8)	66.15%	87.21%	92.14%	53.19%	72.60%
BRAF^V600E^ (-)	132 (33.8)	150 (87.2)					

## Discussion

The BRAFV600E mutation has been extensively studied for its diagnostic and prognostic significance in thyroid cancer, particularly in high-suspicion nodules. Multiple studies have confirmed its role as a molecular marker of malignancy (10–20).

In a 2020 prospective series of 292 patients with high-suspicion thyroid nodules and benign cytology, 36 patients tested positive for BRAFV600E and underwent thyroidectomy. Histopathological examination confirmed papillary thyroid carcinoma (PTC) in 31 cases, corresponding to a specificity of 86.1% and underscoring the utility of the mutation in detecting occult malignancy. The authors recommended routine BRAFV600E testing alongside FNA for nodules with suspicious ultrasound characteristics to enhance diagnostic accuracy (10–20). Similarly, a 10-year follow-up study of 601 PTC patients reported recurrence rates of 18.6% in mutation-positive and 9.9% in mutation-negative cases; multivariable analysis identified BRAFV600E as an independent predictor of recurrence (HR = 2.81, p = 0.006). A large meta-analysis of more than 20,000 patients also demonstrated strong associations between BRAFV600E positivity and adverse clinicopathological factors, including extrathyroidal extension, advanced stage, lymph node metastasis, and recurrence, reinforcing its link to more aggressive disease behavior (14–20).

Our focus on Bethesda II and III nodules reflects their specific clinical challenge: in these categories, particularly when ultrasound is highly suspicious, the main concern is missed papillary thyroid carcinoma (PTC), for which BRAFV600E is a highly specific molecular marker. The present study further supports the clinical relevance of BRAFV600E testing in high-suspicion thyroid nodules, particularly when cytology appears benign or indeterminate despite concerning ultrasound findings. In our series, the prevalence of PTC among C−TIRADS high-suspicion nodules with Bethesda II–III cytology was 69.4%, reflecting a highly selected, surgically treated cohort. The overall malignancy rate of 69.4% reported in this study reflects the entire cohort of surgically treated C-TIRADS high-suspicion nodules with Bethesda II–III cytology. When stratified by cytologic category, malignancy rates were 69.5% for Bethesda II nodules and 91.5% for Bethesda III nodules.

Within this context, ultrasound alone identifies a high-risk group; theoretically, treating all nodules as malignant would yield an apparent accuracy approximating the observed prevalence. However, this approach would sacrifice specificity, as nearly one-third of nodules (30.6%) were benign at final histology, leading to overtreatment. Conversely, BRAFV600E testing demonstrated high specificity (87.2%) and positive predictive value (92.1%), allowing more precise rule-in of malignancy within an already high-risk subset.

Importantly, BRAFV600E positivity was the only independent predictor of PTC on multivariable analysis, whereas individual ultrasound features and composite high-suspicion classification lost significance after adjustment. This finding emphasizes that, once a nodule meets high-suspicion C−TIRADS criteria, molecular data add incremental discriminatory power beyond morphology alone. Clinically, BRAFV600E testing helps differentiate true malignancies from benign mimics among highly suspicious nodules, refining rather than replacing ultrasound-based assessment. In Bethesda III nodules with nuclear atypia, the underlying malignancy is predominantly PTC, making BRAFV600E an appropriate and efficient rule-in test that substantially refines risk stratification in this subgroup.

Consistent with prior reports, BRAFV600E-negative PTCs in our cohort were more likely to exhibit follicular growth patterns or represent less aggressive variants, explaining the moderate sensitivity and highlighting histologic heterogeneity as a source of false-negative results. Univariate analysis revealed no significant differences in age or sex between mutation-positive and mutation-negative groups (p > 0.05). Notably, mutation-positive nodules were smaller (median 0.49 cm vs 0.64 cm, p < 0.001), indicating that even subcentimeter nodules can harbor aggressive molecular alterations. In our series, 93% of papillary thyroid carcinomas were papillary thyroid microcarcinomas (PTMCs, ≤1 cm), reflecting a highly selected cohort of C−TIRADS high−suspicion nodules that had already been triaged to surgery by a multidisciplinary team, rather than the broader population of incidentally detected PTMCs. This predominance of microcarcinomas has two important implications: first, it limits the generalizability of our findings to patients with larger, clinically overt tumors; second, it underscores that BRAFV600E testing in this context primarily serves as a diagnostic ‘rule−in’ tool in nodules already considered for surgery, rather than as a universal criterion to treat all sub-centimeter lesions. Given that many PTMCs are suitable for active surveillance, BRAFV600E positivity alone should not automatically mandate surgical intervention in every small nodule; instead, mutation status should be integrated with ultrasound characteristics, clinical risk factors, and patient preferences when individualizing management.

Although PTMCs are typically considered low-risk tumors suitable for active surveillance, their predominance in this cohort may explain the high malignancy rate and limits generalizability, as many such lesions would not be surgically treated under current ATA guidelines. Thus, caution is warranted when extrapolating these findings to populations with larger or more clinically significant tumors. In our series, the prevalence of papillary thyroid carcinoma among C-TIRADS high-suspicion nodules with Bethesda II–III cytology approached 60–70%. This value reflects the highly selected nature of a surgically treated, ultrasound high-suspicion cohort and should not be interpreted as the baseline malignancy risk of all Bethesda II and III nodules. This finding helps explain the apparent discrepancy with large cytology-based studies reporting malignancy risks of approximately 4% for Bethesda II and 22% for Bethesda III, which are derived from unselected populations including many low-risk nodules managed non-operatively. Our data therefore illustrate how, in carefully selected high-risk clinical and sonographic settings, the true probability of papillary thyroid carcinoma in cytologically benign or indeterminate nodules may substantially exceed the average risks reported in general Bethesda II–III cohorts. Taken together, these considerations highlight that the elevated malignancy rate observed in our cohort is primarily driven by selection and referral patterns intrinsic to a tertiary surgical center, rather than by an inherently higher baseline risk across all Bethesda II and III nodules.

Consistent with our findings, a recent Chinese surgical series of cytologically benign nodules reported a high rate of papillary thyroid carcinoma, further supporting that benign or indeterminate cytology does not exclude malignancy in carefully selected high-risk nodules. These convergent observations underscore the importance of maintaining clinical vigilance when ultrasound features and overall risk profile remain worrisome despite non-malignant FNA results.

As expected in a population with a high pretest probability of PTC (69.4%), the negative predictive value (NPV) of BRAFV600E testing was modest (53.2%), whereas the positive predictive value (PPV) was very high (92.1%). Consequently, a negative result cannot reliably exclude malignancy and should not defer surgery in ultrasound–high-suspicion nodules. Rather, the primary clinical role of this assay lies in “ruling in” cancer: a positive BRAFV600E result increases the post-test probability of malignancy from approximately 70% to over 90% and was associated with more than a 10-fold higher odds of PTC in multivariable analysis. In clinical practice, this information supports recommending surgery more strongly, particularly for patients hesitant about operative treatment or those otherwise eligible for active surveillance based on small tumor size, comorbidity, or preference. Conversely, in BRAFV600E-negative nodules, surgery was still frequently performed due to persistent ultrasound suspicion; however, counseling emphasized the residual diagnostic uncertainty and higher likelihood of benign disease, factors that may influence the extent of surgery and timing of intervention.

Ultrasound analysis confirmed that mutation-positive nodules were more likely to exhibit malignant sonographic features – hypoechogenicity, solid composition, irregular or ill-defined margins, and a taller-than-wide shape – consistent with previous studies linking BRAFV600E to aggressive imaging phenotypes. Microcalcifications (<1 mm), however, did not correlate with mutation status (p > 0.05), suggesting limited predictive value for BRAF positivity. Among the 562 nodules classified as benign on cytology but surgically resected, the malignancy rate remained high (69.4%), illustrating the limitations of FNA alone and underscoring the benefit of adjunctive molecular testing in this challenging subgroup (14–20).

Multivariate logistic regression identified BRAFV600E as the only independent predictor of malignancy (OR = 10.36, p < 0.001). The assay demonstrated a sensitivity of 66.2%, specificity of 87.2%, positive predictive value of 92.1%, negative predictive value of 53.2%, and overall accuracy of 72.6%. Thus, BRAFV600E testing provides moderate sensitivity but high specificity, confirming its value as a “rule-in” rather than “rule-out” diagnostic tool.

Previous research also supports combining BRAFV600E with other molecular and clinical parameters. In older patients, the presence of the mutation substantially increases malignancy risk, even when FNA and ultrasound findings appear benign, highlighting the importance of age-specific risk assessment. Moreover, multigene panels that include BRAFV600E have shown improved diagnostic accuracy and support precision medicine strategies (14–20). Conversely, Bethesda IV nodules are enriched for follicular and RAS-like tumors, where BRAFV600E is uncommon and single-gene testing would be expected to have low yield; therefore, multi-gene panels including RAS and other markers are generally preferred in this setting and fell outside the scope of the present protocol.

Although the sensitivity of BRAFV600E (66.2%) was numerically close to the malignancy prevalence (69.4%), it remained lower than the crude sensitivity implied by treating all high-suspicion nodules as malignant. This difference reflects the complementary roles of ultrasound and molecular testing: ultrasound serves as a gatekeeper for identifying a high-risk group but remains prone to false positives, while BRAFV600E adds specificity and statistical independence in predicting malignancy. In practice, ultrasound should guide biopsy and surgical consideration, whereas BRAFV600E results refine estimated risk within that subset. A positive result materially strengthens the indication for surgery, while a negative result supports conservative counseling but does not automatically defer surgery when ultrasound findings remain highly suspicious.

From a health system standpoint, routine BRAFV600E testing in this narrowly defined indication appears most cost-effective when results are explicitly integrated into shared decision-making – reinforcing surgical recommendation when positive and moderating surgical aggressiveness when negative. Given that testing often requires out-of-pocket payment, its use should remain targeted to carefully selected high-suspicion nodules where the result is likely to meaningfully influence management decisions.

Commercial multigene panels such as Afirma GSC and ThyroSeq v3 have demonstrated high sensitivity and improved overall diagnostic accuracy in indeterminate nodules; however, their high cost and limited availability may restrict widespread adoption in many health care systems.

By contrast, single-gene BRAFV600E testing is inexpensive, technically straightforward, and widely implementable, but primarily functions as a high-specificity “rule-in” test rather than a comprehensive molecular classifier.

The present study was not designed as a direct comparison between BRAFV600E testing and multigene platforms. Future studies incorporating formal cost-effectiveness analyses will be needed to define the most efficient integration of ultrasound, cytology, and single- versus multigene molecular testing across different clinical and economic contexts.

### Limitations

This study has several limitations. The high baseline malignancy rate (69.4%) and the predominance of microcarcinomas (93%) in this surgically treated, ultrasound high-suspicion cohort substantially limit generalizability, and the predictive values reported here should not be extrapolated to lower-risk or unselected Bethesda II–III populations.

As a tertiary referral center, many patients with benign cytology and/or negative or low-risk molecular results are discharged to local hospitals for surveillance. Long-term follow-up data on ultrasound growth, repeat FNA, or delayed surgery were therefore not systematically available for the non-surgical cohort. Given this fragmented follow-up, we could not reliably assess the natural history of BRAFV600E-positive or -negative nodules that did not undergo immediate surgery, nor quantify how often molecular testing led to an ‘upgrade’ from conservative management to operative treatment over time. This lack of a complete denominator and longitudinal follow-up data precluded a formal evaluation of how many cases were ‘ruled in’ for surgery by BRAFV600E testing and prevented a robust cost-effectiveness analysis of routine testing in Bethesda II–III nodules.

The diagnostic role of BRAFV600E in indeterminate or discordant thyroid nodules is well established, and our results are consistent with previous evidence showing moderate sensitivity and high specificity for papillary thyroid carcinoma (PTC). The main contribution of this work is its focus on a narrowly defined but clinically challenging subgroup: C-TIRADS high-suspicion nodules with Bethesda II–III cytology, all of which underwent surgery, allowing complete histologic verification. In this population, BRAFV600E positivity was the strongest independent predictor of malignancy, providing substantial rule-in value even when sonographic features were already highly suspicious. While this design enhances internal validity, it limits external generalizability. One important limitation of our work is the markedly elevated malignancy rate (approximately 70%) observed in this highly selected, surgically treated cohort of C-TIRADS high-suspicion Bethesda II–III nodules. This reflects referral and selection bias and implies that the predictive values of BRAFV600E testing reported here cannot be directly extrapolated to unselected populations with a lower pretest probability of cancer. Additionally, this study is subject to partial verification bias, as only nodules that proceeded to surgery were included in the analysis, while patients with benign cytology, reassuring ultrasound and clinical features, and/or negative BRAFV600E results who were managed conservatively were not systematically evaluated. This selective verification likely enriches the cohort for malignancy and may overestimate specificity and positive predictive value. Therefore, the reported diagnostic performance of BRAFV600E should be interpreted within the context of a surgically treated, high-risk population. Future prospective studies that systematically capture both surgical and non-surgical Bethesda II–III nodules, ideally summarized in a detailed flow diagram, will be necessary to minimize verification bias and provide more generalizable estimates of BRAFV600E test performance.

The exclusive inclusion of surgically treated patients introduces potential selection and spectrum bias, likely inflating performance metrics such as positive predictive value. The lack of a non-surgical comparison cohort and absence of external validation further constrain the broader applicability of these findings. Finally, the single-center design may limit extrapolation to other institutions or patient populations. Despite these limitations, the large sample size and prospective design enhance the reliability of the observed associations. These findings should be considered hypothesis-generating for centers with similar clinical practices, and future multicenter studies incorporating non-surgically managed nodules and expanded molecular panels are needed to better define the clinical utility of BRAFV600E testing in this context. All ultrasound classifications, cytologic diagnoses, and final histopathologic assessments in this study underwent structured multi-reader review with senior expert verification. Although quantitative interobserver agreement metrics were not prospectively collected, the use of consensus-based final diagnoses and blinding of pathologists to BRAFV600E status support the robustness of the diagnostic reference standard applied in our analyses.

Future prospective, multi-center studies with standardized recording of all tested nodules, systematic follow-up of non-surgical patients, and integration of clinical, ultrasound, cytologic, and molecular variables will be essential to quantify the true upgrade rate, long-term outcomes, and cost-effectiveness of BRAFV600E testing in this setting. Additionally, future studies specifically designed to compare outcomes of BRAFV600E-positive versus −negative PTMCs managed with surgery versus active surveillance will be essential to determine whether, and to what extent, BRAFV600E status should modify management recommendations in microcarcinomas compared with larger PTCs.

## Conclusion

BRAFV600E mutation testing meaningfully refines risk stratification among ultrasound–high-suspicion thyroid nodules with benign or indeterminate cytology. It serves primarily as a specific rule-in marker for papillary thyroid carcinoma (PTC) rather than as a broad screening tool. Although the incremental diagnostic gain over ultrasound alone may appear modest at the population level, its impact on individual patient counseling and surgical decision-making within this diagnostic gray zone supports its selective integration into multidisciplinary practice, particularly where molecular testing is available and costs are acceptable. Given its high specificity and positive predictive value, BRAFV600E testing is valuable for confirming malignancy in suspicious nodules. However, because of its moderate sensitivity and limited negative predictive value, it should not be used as a standalone method to exclude cancer. Therefore, the combined interpretation of ultrasound, cytology, and BRAFV600E status remains the most effective approach for risk stratification and management. Ultrasound identifies the high-risk group, while BRAFV600E testing further refines the probability of malignancy within this group – enhancing diagnostic accuracy and supporting timely, individualized management decisions.

## Data Availability

The raw data supporting the conclusions of this article will be made available by the authors, without undue reservation.
