# Supplementary material for: When cytology misses and ultrasound warns: the predictive role of BRAFV600E mutation in thyroid nodules
Source: Front Endocrinol (Lausanne). 2026 Mar 30;17:1803314. doi: 10.3389/fendo.2026.1803314 (PMC13070763; doi:10.3389/fendo.2026.1803314)
Supplement: Supplementary file 1 [file Table1.docx]

**Supplement 1 : Comparison of clinical features between Bethesda II mutation-positive and mutation-negative patients with benign FNA nodules**

| **Features** | **BRAF（-）**  **（n=227）** | **BRAF（+）**  **（n=124）** |  | **P** |
| --- | --- | --- | --- | --- |
| Gender |  |  |  |  |
| Male | 40（17.6） | 21（16.9） | 0.026 | 0.871 |
| Female | 187（82.4） | 103（83.1） |  |  |
| Age |  |  |  |  |
| ＜45 | 100（44.1） | 59（47.6） | 0.403 | 0.526 |
| ≥45 | 127（55.9） | 65（52.4） |  |  |
| ＜55 | 163（71.8） | 102（82.3） | 4.736 | **0.030** |
| ≥55 | 64（28.2） | 22（17.7） |  |  |
| Size（cm） | 0.65（0.46-0.99） | 0.50（0.38-0.70） | -4.371 | **＜0.001** |
| ＜1 | 172（75.8） | 112（90.3） | 10.995 | **＜0.001** |
| ≥1 | 55（24.2） | 12（9.7） |  |  |
| US hypoechoic |  |  |  |  |
| （+） | 194（85.5） | 115（92.7） | 4.034 | **0.045** |
| （-） | 33（14.5） | 9（7.3） |  |  |
| US solid |  |  |  |  |
| （+） | 127（55.9） | 87（70.2） | 6.809 | **0.009** |
| （-） | 100（44.1） | 37（29.8） |  |  |
| US Tall/Wide > 1 |  |  |  |  |
| （+） | 72（31.7） | 80（64.5） | 35.136 | **＜0.001** |
| （-） | 155（68.3） | 44（35.5） |  |  |
| US <1mm calcification |  |  |  |  |
| （+） | 46（20.3） | 24（19.4） | 0.042 | 0.838 |
| （-） | 181（79.7） | 100（80.6） |  |  |
| US irregular borders |  |  |  |  |
| （+） | 149（65.6） | 111（89.5） | 23.807 | **＜0.001** |
| （-） | 78（34.4） | 13（10.5） |  |  |
| No. atypical US findings |  |  |  |  |
| 0 | 24（10.6） | 4（3.2） | 34.301 | **＜0.001** |
| 1 | 27（11.9） | 5（4.0） |  |  |
| 2 | 46（20.3） | 14（11.3） |  |  |
| 3 | 63（27.8） | 28（22.6） |  |  |
| 4 | 55（24.2） | 65（52.4） |  |  |
| 5 | 12（5.3） | 8（6.5） |  |  |

**Supplement 2 : Comparison of clinical features between Bethesda III mutation-positive and mutation-negative patients with benign FNA nodules**

| **Features** | **BRAF（-）**  **（n=55）** | **BRAF（+）**  **（n=156）** |  | **P** |
| --- | --- | --- | --- | --- |
| Gender |  |  |  |  |
| Female | 43（78.2） | 139（89.1） | 4.091 | **0.043** |
| Male | 12（21.8） | 17（10.9） |  |  |
| Age |  |  |  |  |
| ＜45 | 25（45.5） | 74（47.4） | 0.064 | 0.800 |
| ≥45 | 30（54.5） | 82（52.6） |  |  |
| ＜55 | 43（78.2） | 125（80.1） | 0.095 | 0.758 |
| ≥55 | 12（21.8） | 31（19.9） |  |  |
| Size（cm） | 0.52（0.40-0.79） | 0.48（0.39-0.64） | -1.773 | 0.076 |
| ＜1 | 48（87.3） | 148（94.9） | 3.556 | 0.059 |
| ≥1 | 7（12.7） | 8（5.1） |  |  |
| US hypoechoic |  |  |  |  |
| （-） | 4（7.3） | 4（2.6） | 2.472 | 0.116 |
| （+） | 51（92.7） | 152（97.4） |  |  |
| US solid |  |  |  |  |
| （-） | 19（34.5） | 56（35.9） | 0.032 | 0.857 |
| （+） | 36（65.5） | 100（64.1） |  |  |
| US Tall/Wide > 1 |  |  |  |  |
| （-） | 25（45.5） | 63（40.4） | 0.430 | 0.512 |
| （+） | 30（54.5） | 93（59.6） |  |  |
| US <1mm calcification |  |  |  |  |
| （-） | 37（67.3） | 118（75.6） | 1.461 | 0.227 |
| （+） | 181（32.7） | 38（24.4） |  |  |
| US irregular borders |  |  |  |  |
| （+） | 7（12.7） | 16（10.3） | 0.256 | 0.613 |
| （-） | 48（87.3） | 140（89.7） |  |  |
| No. atypical US findings |  |  |  |  |
| 0 | 1（1.8） | 3（1.9） | **6.173** | **0.290** |
| 1 | 3（5.5） | 3（1.9） |  |  |
| 2 | 5（9.1） | 23（14.7） |  |  |
| 3 | 17（30.9） | 53（34.0） |  |  |
| 4 | 26（47.3） | 55（35.3） |  |  |
| 5 | 3（5.5） | 19（12.2） |  |  |

**Supplement 3 :** Comparison of FNA III Benign Nodule Clinical Characteristics Between PTC Group and Benign group

| **Features** | **Benign nodule**  **（n=18)** | **PTC**  **（n=193）** |  | **P** |
| --- | --- | --- | --- | --- |
| Gender |  |  |  |  |
| Female | 14（77.8） | 168（87.0） | 1.193 | 0.275 |
| Male | 4（22.2） | 25（13.0） |  |  |
| Age | 47（36-56） | 46（37-52） | -0.366 | 0.715 |
| ＜45 | 7（38.9） | 92（47.7） | 0.510 | 0.475 |
| ≥45 | 11（61.1） | 209（52.3） |  |  |
| ＜55 | 12（66.7） | 156（80.8） | 2.035 | 0.154 |
| ≥55 | 6（33.3） | 37（19.2） |  |  |
| Size（cm） | 0.51（0.44-0.72） | 0.49（0.39-0.65） | -0.723 | 0.470 |
| ＜1 | 17（94.4） | 179（92.7） | 0.072 | 0.789 |
| ≥1 | 1（5.6） | 14（7.3） |  |  |
| US hypoechoic |  |  |  |  |
| （-） | 3（16.7） | 5（2.6） | 8.943 | **0.003** |
| （+） | 15（83.3） | 188（97.4） |  |  |
| US solid |  |  |  |  |
| （-） | 10（55.6） | 65（33.7） | 3.439 | 0.064 |
| （+） | 8（44.4） | 128（66.3） |  |  |
| US Tall/Wide > 1 |  |  |  |  |
| （-） | 10（55.6） | 78（40.4） | 1.553 | 0.213 |
| （+） | 8（44.4） | 115（59.6） |  |  |
| US <1mm calcification |  |  |  |  |
| （-） | 11（61.1） | 144（74.6） | 1.539 | 0.215 |
| （+） | 7（38.9） | 49（25.4） |  |  |
| US irregular borders |  |  |  |  |
| （-） | 2（11.1） | 21（10.9） | 0.001 | 0.976 |
| （+） | 16（88.9） | 172（89.1） |  |  |
| BRAFV600E |  |  |  |  |
| （-） | 13（72.2） | 42（21.8） | 21.754 | **＜0.001** |
| （+） | 5（27.8） | 151（78.2） |  |  |

**Supplement 4 :** Comparison of FNA II Benign Nodule Clinical Characteristics Between PTC Group and Benign group

| **Features** | **Benign nodule**  **（n=154)** | **PTC**  **（n=197）** |  | **P** |
| --- | --- | --- | --- | --- |
| Gender |  |  |  |  |
| Female | 126（81.8） | 164（83.2） | 0.123 | 0.726 |
| Male | 28（18.2） | 33（16.8） |  |  |
| Age | 48（39-55） | 46（41-53） | -0.298 | 0.766 |
| ＜45 | 70（45.5） | 89（45.2） | 0.003 | 0.959 |
| ≥45 | 84（54.5） | 108（54.8） |  |  |
| ＜55 | 111（72.1） | 154（78.2） | 1.736 | 0.188 |
| ≥55 | 43（27.9） | 43（21.8） |  |  |
| Size（cm） | 0.74（0.48-1.14） | 0.51（0.38-070） | -6.067 | **0.001** |
| ＜1 | 103（66.9） | 181（91.9） | 34.963 | **＜0.001** |
| ≥1 | 51（33.1） | 16（8.1） |  |  |
| US hypoechoic |  |  |  |  |
| （-） | 27（17.5） | 15（7.6） | 8.072 | **0.004** |
| （+） | 127（82.5） | 182（92.4） |  |  |
| US solid |  |  |  |  |
| （-） | 76（49.4） | 61（31.0） | 12.278 | **＜0.001** |
| （+） | 78（50.6） | 136（69.0） |  |  |
| US Tall/Wide > 1 |  |  |  |  |
| （-） | 110（71.4） | 89（45.2） | 24.260 | **＜0.001** |
| （+） | 44（28.6） | 108（54.8） |  |  |
| US <1mm calcification |  |  |  |  |
| （-） | 126（81.8） | 155（78.7） | 0.533 | 0.465 |
| （+） | 28（18.2） | 42（21.3） |  |  |
| US irregular borders |  |  |  |  |
| （-） | 60（39.0） | 31（15.7） | 24.277 | **＜0.001** |
| （+） | 94（61.0） | 166（4.3） |  |  |
| BRAFV600E |  |  |  |  |
| （-） | 137（89.0） | 90（45.7） | 70.849 | **＜0.001** |
| （+） | 5（11.0） | 107（54.3） |  |  |

**Supplement 5 :**PTC Risk in FNA II Benign Nodules by Multivariable Logistic Regression Analysis

| **Features** | B | OR（95%CI） | P |
| --- | --- | --- | --- |
| Size | -0.973 | 0.378（0.126-1.131） | 0.082 |
| Size≥1cm | -0.581 | 0.559（0.177-1.769） | 0.323 |
| US hypoechoic | -0.210 | 0.811（0.305-2.151） | 0.673 |
| US solid | 0.361 | 1.435（0.824-2.498） | 0.202 |
| Tall/Wide > 1 | 0.203 | 1.225（0.693-2.165） | 0.485 |
| US irregular borders | 0.499 | 1.648（0.804-3.375） | 0.172 |
| BRAF mutation | 1.986 | 7.290（3.917-13.568） | **＜0.001** |

**Supplement 6 :**PTC Risk in FNA III Benign Nodules by Multivariable Logistic Regression Analysis

| **Features** | B | OR（95%CI） | P |
| --- | --- | --- | --- |
| US hypoechoic | 1.755 | 5.786（1.044-32.076） | 0.045 |
| BRAF mutation | 2.171 | 8.769（2.914-26.389） | **＜0.001** |

**Supplement 7 :**Comparing BRAFV600E mutation testing results with postoperative pathologic findings in Bethesda class III subgroups

|  | **PTC** | **benign nodule** | **Sensitivity** | **Specificity** | **PPV** | **NPV** | **Accuracy** |
| --- | --- | --- | --- | --- | --- | --- | --- |
| BRAF^V600E^（+） | 151（78.2） | 5（27.8） | 78.2% | 72.2% | 96.80% | 23.64% | 77.73% |
| BRAF^V600E^（-） | 42（21.8） | 13（72.2） |  |  |  |  |  |
